# BioSCOOP – Biobank Sample Communication Protocol. New approach for the transfer of information between biobanks

**DOI:** 10.1093/database/baz105

**Published:** 2019-10-11

**Authors:** J Jarczak, J Lach, P Borówka, M Gałka, M Bućko, B Marciniak, D Strapagiel

**Affiliations:** 1 Biobank Lab, Department of Molecular Biophysics, Faculty of Biology and Environmental Protection, University of Łódź, Łódź, Poland; 2 BBMRI.pl Consortium, Wrocław, Poland; 3 Department of Anthropology, Faculty of Biology and Environmental Protection, University of Łódź, Łódź, Poland; 4 Independent IT Specialist; 5 Bee2code sp. z o.o., ul. Daszyńskiego 5; 44-100 Gliwice

## Abstract

Dynamic development of biobanking industry (both business and science) resulted in an increased number of IT systems for samples and data management. The most difficult and complicated case for the biobanking community was cooperation between institutions, equipped with different IT systems, in the field of scientific research, mainly data interchange and information flow. Tools available on the market relate mainly to the biobank or collection level. Efficient and universal protocols including the detailed information about the donor and the sample are still very limited. Here, we have developed BioSCOOP, a communication protocol in the form of a well documented JSON API. The main aim of this study was to harmonize and standardize the rules of communication between biobanks on the level of information about the donor together with information about the sample. The purpose was to create a communication protocol for two applications: to transfer the information between different biobanks and to allow the searching and presentation of the sample and data sets.

## Introduction

Every organization, sooner or later will have to face the problem of difficulties in communication. A precise information flow is crucial and allows for quick decision making, prevents conflicts, and facilitates daily work. The lack of communication hinders current work and leads to wasting time. In the world of biobanks, information flow and data transfer are the basis of efficient functioning of units which were created to collect biological material for further advanced research. Dynamic development of biobanking industry (both business and science) resulted in an increased number of IT systems for samples and data management. The most difficult and complicated case for biobanking community was the cooperation between institutions, equipped with different IT systems, especially in the field of data harmonisation (data interchange and information flow). The problem starts when we want to describe parameter when different scales are commonly used. Temperature can be an excellent example. It can be measured in at least three different scales (Kelvin, Fahrenheit and Celsius), which refer to the same value but are described using three different units. Exchange of information about temperature between two entities using different scales can lead to misunderstanding. Biobanks are invaluable sources of data with huge potential of biological material reuse, sometimes limited by communication restrictions resulted from heterogeneity of biorepositories (1–3). In their repositories, they store, sample sets that are supplemented by the large data sets composed of (depending on the type of biobank): the list of phenotypic features of the donor, information about the diseases and all medical history, lifestyle information, information about the sample, information about storage and quality parameters etc. For researchers, any information about the collected samples and data is extremely important in connection to the data they generate. The need to conduct research on a very specific and precisely defined sample set is undeniable. In the era of big data and increasing importance of personalized medicine, data visibility and access, storage, management and integration has become a major issue in biobanking and biomedical research (4). Increasing number of specialized biorepositories and expansion of available data types produced by biomedical or research centres, require adequate sample information and management systems (e.g. BIMS, BBMS, LIMS) for location and integration of metadata, along with well-defined sample description standards for stored biological material (e.g. ICD-10, SPREC, BRISQ) (5–9). Due to varying specific features of biomedical facilities or biobanks worldwide, IT solutions for sample information and management are often tailor-made for biorepositories, stemming directly from the type of basic research, used biological material, storage requirements or survey restrictions (5). These internal standards become a challenge for biobank- to biobank communication or data exchange throughout biobanking networks, primarily created for facilitation of data interchange. Direct communication between biobanks, which are providers of biological material for secondary research (in accordance with the Tri-Council Policy Statement (10)) is troublesome due to tree key limitations: divergent description of the samples, different levels of accuracy about the donor and incompatible IT solutions for data storage and transfer (10,11). Currently, there are many standards for sample management implemented by biorepositories which touch upon issues of donor-sample description (12–14), sample SOPs (15–17), directories of biological material collections (16,18), ontology of collections and biobanks (18–29), network and integration protocols for biobanks (30–36), or even biobank-biobank matching algorithms (37). These factors reflect the complexity of unification of individual sample description for communication/exchange protocol between biobanks. Universal FOSS (free and open source software) or protocols containing minimal information about sample-donor operating on common communication IT infrastructure, are still very limited. However, there are attempts to improve communication between biobanks and first solutions to facilitate sample location and access such as service Negotiator 1.0 made by BBMRI-ERIC (38) and Sample Request Portal (open source portal PODIUM) prepared by BBMRI-nl (national node of BBMRI-ERIC in Netherlanden). The proposed standard joins different ontologies used in sample-donor description models such as MIABIS, BRISQ etc., use recognised disease ontologies e.g. ICD-10, with parameters used in ergonomics, anthropometry and biomechanics e.g. ISO-TC159/SC3: therefore, it collectively provides effective networking and resource sharing between biobanks. The main aim of this study was to harmonize and standardize the rules of communication between biobanks on the level of information about the donor. To address these issues, BioSCOOP was created as a communication protocol for two applications: to transfer the information between different biobanks and to allow searching and presentation of sample and data sets.

## Results

BioSCOOP has the form of a well documented JSON API which describes an organized data format for a list of attributes describing the donor with particular emphasis on the phenotype, anthropological measurements, medical data and sample material. The software application of this standard was created using Swagger Editor, a tool for API creation, to be compliant with Open API Specification. BioSCOOP has been deposed on Github, as YAML file and can be easily imported into Swagger Editor or any other text editor as a described JSON.

Furthermore, an exemplary data set has also been prepared. It can be downloaded and used for test sample search using the proposed browser – Bioface. It was provided to guide users through sample search based on BioSCOOP standard.

The list of features included in BioSCOOP is listed in Table S1 (supplementary information).

### Implementation

Import of data in BioSCOOP format has been implemented in the related project, Bioface. Bioface has a distributed architecture and is designed as a browser for the members of Polish Biobanking Network (PBN) (39) as well as a broader group of biobanks and researchers, in order to search for samples from different biobanks and biorepositories. It is a part of IT infrastructure for PBN, which includes both central and distributed solutions for data collection and sharing. Implementation was divided into three independent steps:

1. Test data set preparation – an exemplary data set was prepared using Microsoft Excel spreadsheet. It contains randomly generated information about 200 database records mimicking samples collected from 200 mock donors. The provided information includes: birth date, place of birth and residence, sex, ethnic origin, skin tone, hair and eye colour, blood group, parameters like WHR (waist hip ratio), BMI (body mass index), CI (Corpulence index), some of anthropological features, diseases and medical procedures undergone by the donor and form of sample material (Tab. S1.). This information was supplemented by donor ID, collection ID, sample ID and measurement/event date timestamp. The format includes also information on the source of included data (donor questionnaire). This test file was initially prepared in.csv format. Then, the data set has been transformed with the use of a homemade script written in Python. This script, by imported data from.csv file, and converted them into JSON, according to the data format written in BioSCOOP.
2. Registration in Bioface – this step was necessary to carry out the sample search procedure. For testing purposes, we first created a dummy account with a mock biobank in Bioface. We subsequently uploaded the previously generated JSON-format data set and used it to perform test searches of the included mock samples.
3. Sample search – various queries have been tested to obtain defined sample set. Queries structure is characteristic for Apache Solr search platform which is a base of Bioface.

Examples of basic queries structure:

a. Basic queries:

field_name:value; e.g. gender:male

b. Phrase query:

field_name:“string value”; e.g. birthPlace:“Gdansk, Poland”

c. Range query:

numeric_field_name:[lower_limit TO upper_limit]; e.g. bmi:[18 TO 23]

Also using logical operators to combine subsequent parts of query is posible. The above examples do not exhaust the possibility of creating queries in the used engine, which are described in more detail in the Apache Solr documentation (https://lucene.apache.org/solr/guide/).

## Conclusions and future developments

BioSCOOP was created as a communication protocol and aims to facilitate and improve the information transfer in a large network of biobanks. The members of the Polish Biobanking Network will be involved in first implementation of described protocol. On the basis of this, there are further goals such as gathering specialists in many fields of science in one workgroup to create the most accurate way for description data collected by biobanks and scientists. We discuss also future developments. The next step is implementation of BioSCOOP in the BIMS system, currently being created by the Polish Biobanking Network. BioSCOOP will also be used as a data import format in data processing IT software developed by the Polish Biobanking Network.

## Supplementary Material

Tab_S1_baz105

## Supplementary data

- Bioscoop available from: http: https://github.com/BiobankLab/BioSCOOP
- Table of features describing the content of Bioscoop – Tab. S1
- Exemplary data set to perform an usability check – file in JSON format https://github.com/BiobankLab/BioSCOOP
- Demo Bioface for testing purpose - available from: http://biobank.uni.lodz.pl:8082/bioface/
